# Compressive Strength and Porosity Evaluation of Innovative Bidirectional Spiral Winding Fiber Reinforced Composites

**DOI:** 10.3390/jcm11226754

**Published:** 2022-11-15

**Authors:** Naji Kharouf, Salvatore Sauro, Louis Hardan, Hamdi Jmal, Gulbahar Bachagha, Valentina Macaluso, Frédéric Addiego, Francesco Inchingolo, Youssef Haikel, Davide Mancino

**Affiliations:** 1 Department of Biomaterials and Bioengineering, INSERM UMR_S 1121, 67000 Strasbourg, France; 2 Department of Endodontics and Conservative Dentistry, Faculty of Dental Medicine, University of Strasbourg, 67000 Strasbourg, France; 3 Dental Biomaterials and Minimally Invasive Dentistry, Department of Dentistry, University CEU Cardenal Herrera, CEU Universities, C/Santiago Ramón y Cajal, s/n, Alfara del Patriarca, 46115 Valencia, Spain; 4 Department Interdisciplinary of Bari, Università di Bari “Aldo Moro”, Giulio Cesare Square, 11, 70124 Bari, Italy; 5 Department of Restorative Dentistry, School of Dentistry, Saint-Joseph University, Beirut 11072180, Lebanon; 6 ICube Laboratory, Mechanics Department, UMR 7357 CNRS, University of Strasbourg, 67000 Strasbourg, France; 7 ESTA, School of Business & Technology, 90000 Belfort, France; 8 Luxembourg Institute of Science and Technology (LIST), Department Materials Research and Technology (MRT), ZAE Robert Steichen, 5 Rue Bommel, Hautcharage, L-4940 Luxembourg, Luxembourg; 9 Pôle de Médecine et Chirurgie Bucco-Dentaire, Hôpital Civil, Hôpitaux Universitaire de Strasbourg, 67000 Strasbourg, France

**Keywords:** bidirectional reinforced fiber composite, bidirectional spiral winding reinforced fiber composite, post-and-core materials, compressive strength

## Abstract

The aim of this in vitro study was to investigate the compressive strength and the bulk porosity of a bidirectional (bFRC) and an experimental bidirectional spiral winding reinforced fiber composite (bswFRC). Cylindrical-shape specimens were prepared for each material group and processed for the evaluation of compressive strength after different storage conditions (dry, 1 and 3 months) in distilled water at 37 °C. The specimens were also assessed for the degree of bulk porosity through X-ray tomography. A scanning electron microscope (SEM) was used to determine the fracture mode after a compressive strength test. Data were statistically analyzed using Two-Way Analysis of Variance (ANOVA). A significantly lower compressive strength was obtained in dry conditions, and after 1 month of water immersion, with the specimens created with bFRC compared to those made with bswFRC (*p* < 0.05). No significant difference (*p* > 0.05) was found between the two groups after 3 months of water immersion. However, the presence of water jeopardized significantly the compressive strength of bswFRC after water storage. The type of fracture was clearly different between the two groups; bswFRC showed a brutal fracture, whilst bFRC demonstrated a shear fracture. The bswFRC demonstrated higher pore volume density than bFRC. In conclusion, bswFRC is characterized by greater compressive strength compared to bFRC in dry conditions, but water-aging can significantly decrease the mechanical properties of such an innovative FRC. Therefore, both the novel bidirectional spiral winding reinforced fiber composites (bswFRC) and the bidirectional fiber reinforced composites (bFRC) might represent suitable materials for the production of post-and-core systems via CAD/CAM technology. These findings suggest that both FRC materials have the potential to strengthen the endodontically treated teeth.

## 1. Introduction

A successful endodontic treatment relies on several technical factors such as good shaping, proper irrigation, and optimal tridimensional filling of the root canal system [1,2,3]. Furthermore, a clinician must be able to choose the most adequate restorative approach to restore endodontically treated teeth (ETT), as these latter become more vulnerable to fracture than vital teeth [4]. Indeed, during endodontic treatments, a great amount of dental tissue may be removed, especially due to the preparation and disinfection steps; this may represent the key cause for the tooth structure to become weak [5,6].

Different types of restorative approaches have been advocated to reconstruct and reinforce ETT. For instance, crowns are often used when the coronal structure is sufficient to provide enough retention. Conversely, fiber or metal posts can be employed to increase the retention of crown to the root canal. Post-and-core systems can adapt very well to root canal morphology, and these are also used to achieve greater aesthetic results in anterior teeth [7,8,9,10]. On the other hand, the purpose of endo-crown restorations is to allow the reconstruction of the root canal system, to replace missing dental tissues, restore coronal morphology and tooth function, as well as to provide the necessary strength to prevent tooth fracture during mastication [11]. Different criteria play an important role in the clinical success rate of restorations, such as the amount of residual coronal structure, the restorative technique, and the materials employed during the treatment [12,13].

Among all the materials that can be used in this field, prefabricated posts do not seem to be properly adapted to residual dental structure, and therefore these cannot be adapted to the size and shape of the root canal properly [9]. Teeth restored using this latter approach are often characterized by voids within the adhesive interface and by a considerable amount of cement required to fill such a lack of adaptation between the post and the root canal walls [9,14]. Unfortunately, a thick layer of the cement may lead to loss of retention, with consequent detachment and failure of the post [15]. Moreover, due to their different moduli of elasticity, post and coronal restoration causes an inhomogeneous distribution of the intra-oral torque forces to the root dentin, with a consequent increase in the risk of fracture [16,17].

The post-and-core (PaC) is a “one-piece” system generated using specific technology such as CAD/CAM in order to obtain a custom-made product, which can adapt well to the morphology of the root canal walls [18,19]. The presence of less cement thickness and voids represent the main advantages of this technique [20].

Several materials can be used to construct PaC systems, such as metal, ceramic or fiberglass [11,19,21,22]. The choice is based on the strength and aesthetic recommendations, and it also depends on the tooth and its location in oral cavity (e.g., posterior or anterior teeth). However, some of these materials, such as metal or ceramic, may increase the risk of fracture in the remaining tooth structure due to their high modulus of elasticity, and they may produce gray discoloration of the crown [18]. Conversely, fiberglass post-and-core systems present a modulus of elasticity similar to that of dentin, which may provide favorable results in terms of biomechanical and aesthetic properties [5,23]. PaC systems can be manufactured in different angulations, sizes, and shapes with high precision and efficiency using CAD/CAM technology [23]. Different structures of fiber-reinforced composites are introduced in the dental market [24].

Recently, bidirectional spiral winding glass fiber reinforced composites have been introduced [25] in dental practice, but there is no study in the literature about such novel materials.

Therefore, the aim of the present study was to evaluate the compressive strength and porosity level of two fiber reinforced composite systems known as bidirectional fiber reinforced composite, “bFRC”, and bidirectional spiral winding fiber reinforced composite, “bswFRC”. The hypothesis of this study was that there would be significant differences between the tested systems.

## 2. Materials and Methods

### 2.1. Materials

Bidirectional fiber reinforced composite “bFRC” and bidirectional spiral winding fiber reinforced composite “bswFRC” (Bio Composants Médicaux, Auvergne-Rhône-Alpes, France) were used in the present study. All specimens were prepared using CAD/CAM equipment “INDEX 35I Pro” (imes-icore GmbH, Eiterfeld, Germany) with WORKNC software (Hexagon, Charnay-les-Mâcon, France) in order to obtain 20 cylinders (4 mm in diameter and 4.5 mm in height) for each group.

### 2.2. X-ray Tomography

The internal structure of the specimens created with bFRC and bswFRC was inspected in 3D by means of micro-computed X-ray tomography (µCT) (EasyTom 160 from RX Solutions, Chavanod, France). Imaging was conducted at a voltage of 45 kV and a current of 160 mA, using a micro-focused tube equipped with a tungsten filament. The source-to-detector distance (SDD) and the source-to-object distance (SOD) were adjusted in such a way to obtain a voxel size of around 2.3 µm. The volume reconstruction was executed through the software Xact64 (RX Solutions) after applying treatments such as geometrical corrections and ring artefact attenuation. The image treatment was performed with Avizo software (ThermoFisher, Waltham, MA, USA) that enabled us to (i) de-noise the images with a median filter, (ii) segmentate the image intensity to reveal the objects of interest (here the pores), (iii) remove insignificant small objects (below a size of 10 pixels) from the segmented 3D data, and (iv) determine the 3D geometrical aspects of the objects of interest (volume and equivalent diameter) [26].

### 2.3. Compressive Strength Test

The specimens (*n* = 15) were submitted to a compressive strength test. Three different periods (0 h “dry conditions”, 1 month, and 3 months) of storage in distilled water at 37 °C were evaluated (5 specimens each period). The specimens were tested using a universal testing machine (Instron Machine 5969, High Wycombe, UK) equipped with a 50 kN load cell, which recorded the load applied to the specimens at a crosshead speed of 0.5 mm/min. The specimens were placed between two steel plates and the compression tests were performed until failure. The values were recorded for the maximum force applied at fracture. The compressive strength was calculated in megapascals (MPa) according to the formula:

*σc* = 4*P*/*πD*^2^

where *P* is the recorded load during the test and *D* is the initial sample diameter.

### 2.4. Scanning Electron Microscopy Observation (SEM) for Fracture Types

After fracture, the specimens were ultrasonically cleaned for 3 min, immersed in 100% ethanol for 2 min, air dried, mounted on metal stubs, and then sputter-coated with a gold–palladium alloy (20/80 wt.%) using a Hummer JR sputtering device (Technics, San Jose, CA, USA). These were analyzed using a Quanta 250 FEG (field emission gun) scanning electron microscope “SEM” (FEI Company, Eindhoven, The Netherlands), with an electron acceleration voltage of 10 kV and a working distance of 10 mm [27] to determine the type of fracture and to observe the direction of the fracture into the fibers.

### 2.5. Statistical Analysis

Data were analyzed with SigmaPlot release 11.2 (Systat Software, Inc., San Jose, CA, USA). Two Way Analysis of Variance including multiple comparison procedures (Holm-Sidak method) was used to determine whether significant differences existed in the compressive strength values. A statistical significance level was set at α = 0.05.

## 3. Results

**Figure 1:**
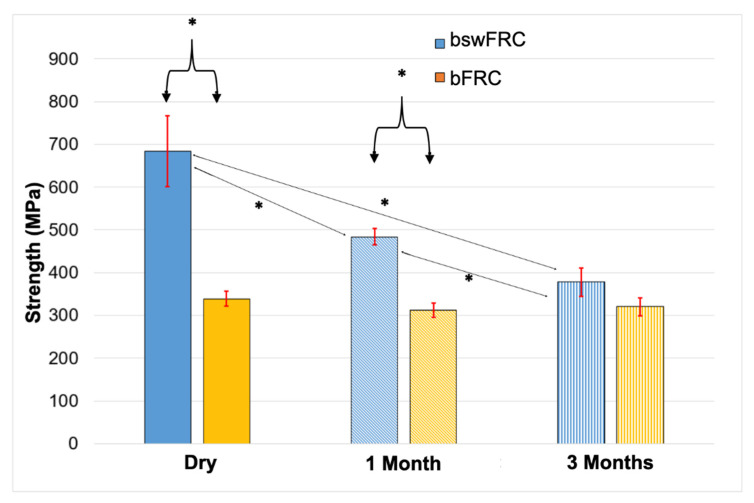
Compressive strength values (mean and standard deviations “MPa”) for bFRC and bswFRC at three aging periods in water at 37 °C (T = 0 “dry conditions”, T = 1 month, T = 3 months). * *p* < 0.05.

A significantly lower compressive strength was obtained for bFRC compared to bswFRC in dry conditions (*p* < 0.05). Moreover, the same tendency was observed at 1 month of immersion in water at 37 °C, where the compressive strength of bFRC was significantly lower than that of the specimens created with bswFRC (*p* < 0.05). In contrast, at 3 months of immersion in water, no statistically significant difference was found between the compressive strength of the two tested groups (*p* > 0.05) (Figure 1 and Table 1). Concerning bswFRC, the compressive strength significantly decreased over time (*p* < 0.05), whilst the compressive strength of bFRC presented no significant difference over the different periods of water storage (*p* > 0.05) (Figure 1 and Table 1).

**Figure 2:**
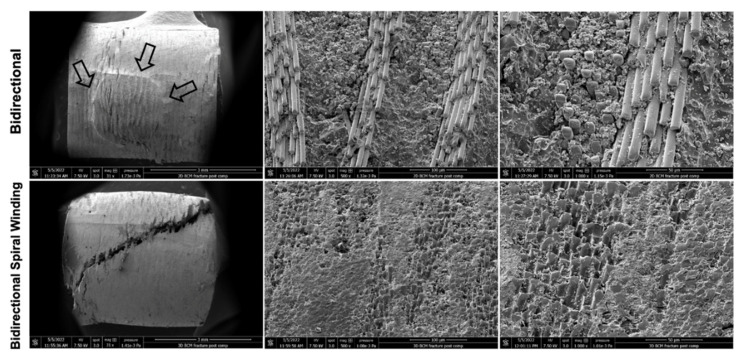
Representative scanning electron microscopy photos of bFRC and bswFRC fractures at different magnifications (×31, ×500 and ×1000). Black arrows indicate a shear fracture in bFRC sample.

Subsequent to the compressive strength test, all the specimens were observed using SEM in order to investigate the type of fracture and the propagation of the fracture in the different composites. The most common fracture observed in the specimens created with bswFRC was an oblique fracture (Figure 2). Conversely, the specimens created with the bFRC presented prevalently a shear fracture in the middle of the bulk material with the exposed fibers (Figure 2, black arrow).

**Figure 3:**
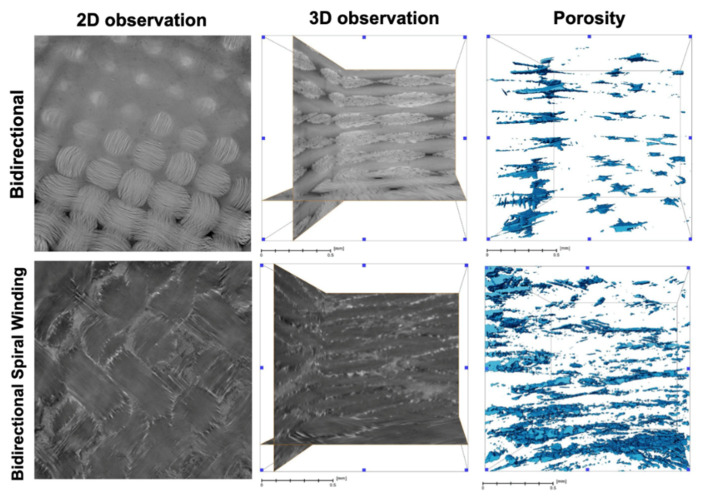
Volume rendering of the segmented pores (blue color) in bFRC and bswFRC, obtained by X-ray tomography analysis. The scale bar corresponds to 0.5 mm in all images.

The X-ray tomography analysis showed that bswFRC material had higher pore volume density than bFRC (Table 2 and Figure 3). In the 3D observations, the resin and fiber layers could be detected and distinguished for bFRC (Figure 3, 3D observation); in contrast, in the specimens created with bswFRC, it was impossible to clearly discriminate such different layers.

## 4. Discussion

Restoration of endodontically-treated teeth with optimal crown retention is still a challenge due to the important loss of dental hard tissues that endodontists usually have to face in seriously caries-compromised teeth [6]. Besides fiber or metallic posts, post-and-core systems (PaC) may represent a suitable restorative solution in such a clinical scenario; they have the advantage of being better adapted to the root canal morphology [11]. Indeed, PaC can be designed and fabricated using different approaches (e.g., CAD/CAM) and materials such as resin with glass fibers (composite), with elastic modulus similar to that of dentin [23].

In the current study, two different types of innovative PaC materials (bidirectional, and bidirectional spiral winding, fiber reinforced composites) were characterized by evaluating their compressive strength and the overall bulk porosity through X-ray tomography. It was interesting to observe the presence of significant differences between both materials, thus, the hypothesis tested in this study must be accepted.

The tested materials were tested in dry conditions and after two different storage periods (1 and 3 months) in distilled water at 37 °C. This aging protocol was used exclusively to stress hydrolytically the tested materials and evaluate their mechanical properties. In clinical situations, these materials are not exposed to saliva or water in the oral cavity, thus, the aging protocol used in the present study may not be a relevant criterion for a clinical scenario. Moreover, the post-and-core part must be protected by the crown and by cement in the root canal; therefore, the presence of water is typically quite rare.

According to the manufacturer, both materials tested in this study have the same percentage of resin (41 wt.%) and glass fiber (59 wt.%) [25]. Therefore, the results of compressive strength could be related to the different arrangement of fiber within the resin matrix of both materials. Indeed, the 3D observation demonstrated no clear resin layer between the glass fiber layers in bswFRC (spiral winding) (Figure 3). In contrast, bFRC showed a net interface between resin and glass fiber layers (Figure 3). A stable fiber–resin interface allows a smooth stress transfer between the material phases (fiber and matrix). Hence, when the stress is uniformly distributed within the material, the material’s strength reaches high and stable values. Conversely, after the water-aging, the stress is not homogenously distributed within the material. We hypothesize that the reason the water-aging affects bswFRC more than bFRC is probably due to the higher porosity of the bswFRC material. This porosity, as well as the water-aging, create zones where stress is more concentrated and the mechanical properties are altered.

As mentioned previously (Figure 1), water had no effect in the specimens in the bFRC group (*p* > 0.05) up to 1 month of storage, while bswFRC demonstrated a decrease in the compressive strength over time in water (*p* < 0.05). No significant difference was found between the compressive strength of both materials after 3 months of immersion in water (*p* > 0.05).

In order to understand these results, X-ray tomography was performed on the specimens of both groups (Figure 3), and it was interesting to observe a higher porosity in bswFRC compared to bFRC. We can hypothesize that the higher porosity of bswFRC could be responsible for greater water uptake within the internal structure of the materials, which in turn jeopardized the mechanical properties. Indeed, as these materials consist principally of resin (urethane dimethacrylate) and glass fiber, it may be possible that the water could have affected the integrity of the interface resin–fiber due to hydrolytic degradation [28,29]. Moreover, Paturel et al. [30] demonstrated that water sorption could affect the properties of similar materials (glass–fiber/resin composite). Water can induce degradation of the resin network and the interface between fiber and resin increases [31].

**Figure 4:**
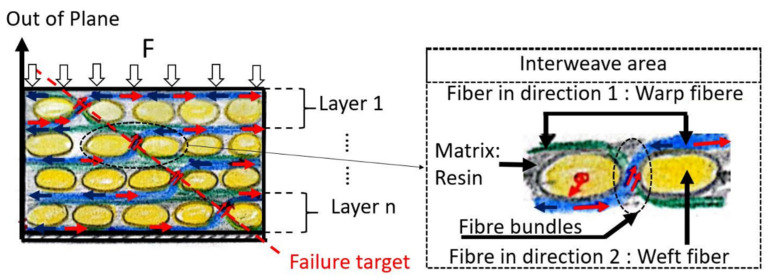
Illustration of out-of-plane compression failure mechanism of woven fibre composite. Red → arrow and blue ← arrow: local normal forces transferred to fibre due to Poisson effect. ↗↗ Double red arrow: Forces in fiber bundles zone which is a combination of shear, tensile and compression forces. ↓ Black arrow: compression-loading force. Dash red line: Failure target. For bswFRC, the mechanical stress concentration is in interweave zone (bundle zone). Thus, the failure target across this bundle zone.

Regarding the fracture mode, the type of fracture was different in bswFRC compared to bFRC. A typical out of plane compression fracture was observed in the specimens created with bswFRC. A brittle longitudinal fracture (45°), without layer displacement in the middle of the cylinders (Figure 2), was reported for bswFRC. For this material, the fibers were probably subjected to a high shear stress due to the characteristic structure in the interweave zone of the composite (Figure 4) [32]. In addition, such fibers may have undergone an unexpected extension due to the Poisson effect [32]. Once the fiber bundles were subject to a tensile or shear failure (Figure 4), the resin in and around the fiber bundles also developed cracks [32].

For bFRC, the crack occurred in the resin layer between the glass–fiber layers. Thus, a shear force between the different layers is observed (slippage between the layers). The crack propagated in the resin layer (more delicate) and a horizontal fracture finally occurred (Figure 2). In addition, the SEM images showed exposed fibers in the middle of the cylinders. As well as for bswFRC, the fibers of bFRC were subjected to tensile–shear loading, but we speculate that the presence of a thick resin layer between fiber-woven layers in the bFRC group may have prevented a proper stress distribution during the compression test, so that the stress concentrated at the resin layer where the fracture occurred.

These findings suggest that in clinical practice, CAD/CAM indirect fiber post-and-cores could be considered as the favorite choice and as clinically promising to restore ETT, due their high fracture strength with less risk of nonrepairable tooth fracture [33]. This technology could be an option for dentists to use digital technology [34] with a CAD/CAM system and materials such as bFRC and bswFRC, which can strengthen ETT. The adaptation of this technology to tooth structure plays an important role in the longevity of tooth restoration, which provides clinical success [35]. Accompanying the evolution of CAD/CAM technology, materials companies continue in the development of stable, esthetic and higher resistance materials in order to attain an optimal restoration using CAD/CAM technology in an oral environment.

Manufacturing post-and-cores via CAD/CAM technology and using bswFRC material is an innovative idea under development for the dental market. Our experimental setup has helped to determine the compressive strength before and after water aging in both materials. Indeed, the values obtained showed a constant load resistance for the bFRC group, and a considerable decrease for the bswFRC group due to water aging.

## 5. Conclusions

The bswFRC presented superior results in compressive strength compared to bFRC in dry conditions, but water-aging can significantly decrease the mechanical properties of such an innovative FRC. Therefore, both the novel bswFRC and bFRC may represent suitable materials for the production of PaC systems via CAD/CAM technology.

However, further studies are recommended to test these materials in teeth, and under different conditions and different thermo-mechanical and hydration aging.

## Figures and Tables

**Table 1:** Evolution of compressive strength (mean ± standard deviations “MPa”) for bFRC and bswFRC in dry conditions, and after immersion in water at 37 °C for 1 month and 3 months. *p* < 0.05.

Group	Dry	1 Month	3 Months	Statistical Analysis (*p* < 0.05)
bFRC	338 ± 19	311 ± 18	321 ± 23	No
bswFRC	684 ± 92	484 ± 21	378 ± 36	Yes
Statistical analysis (*p* < 0.05)	Yes	Yes	No	

**Table 2:** Pore volume density (%) of bFRC and bswFRC as calculated from X-ray tomography imaging.

Group	Pore Volume Density (%)
bFRC	0.60
bswFRC	1.23

## Data Availability

The data presented in this study are available on request from the corresponding author.
